# Taste Modulation by Umami Compounds: Mechanisms, Sensor-Based Evaluation, and Pharmaceutical Applications in Bitterness Suppression

**DOI:** 10.3390/s26165073

**Published:** 2026-08-10

**Authors:** Takahiro Uchida, Takeshi Onodera, Ziyi Jiang, Kiyoshi Toko

**Affiliations:** 1 Food and Health Innovation Center, Nakamura Gakuen University, 5-7-1 Befu, Jonan-ku, Fukuoka 814-0198, Japan; ktoko@nakamura-u.ac.jp; 2 Faculty of Information Science and Electrical Engineering, Kyushu University, 744 Motooka, Fukuoka 819-0395, Japan; onodera@ed.kyushu-u.ac.jp; 3 Graduate School of Information Science and Electrical Engineering, Kyushu University, 744 Motooka, Nishi-ku, Fukuoka 819-0395, Japan; jiang.ziyi.906@kyudai.jp; 4 Graduate School of Nutritional Sciences, Nakamura Gakuen University, 5-7-1 Befu, Jonan-ku, Fukuoka 814-0198, Japan; 5 Institute for Advanced Study, Kyushu University, 744 Motooka, Nishi-ku, Fukuoka 819-0395, Japan

**Keywords:** bitterness suppression, umami substances, taste sensor, taste modulation, TAS2R, oral pharmaceutical palatability

## Abstract

Bitterness of oral active pharmaceutical ingredients (APIs) remains a major barrier to patient adherence, particularly in pediatric and geriatric populations. Conventional taste-masking strategies primarily rely on formulation-based approaches, but do not always sufficiently suppress activation of bitter taste pathways. Increasing attention has focused on taste-modulating compounds, including umami substances, which reduce bitterness through multiple stages of taste processing, including peripheral receptor responses, perceptual taste interactions, and central sensory integration. This review provides an integrated overview of umami-mediated bitterness suppression from molecular, sensory, neural, and analytical perspectives. Interactions between TAS2R bitter receptors and T1R1/T1R3 umami receptors, including possible downstream signaling convergence and sensory integration mechanisms, are discussed in relation to bitterness suppression. Representative umami compounds, including monosodium glutamate (MSG) and umami peptides, have been reported to suppress the bitterness of several bitter APIs, suggesting potential applicability beyond compound-specific taste-masking strategies. Recent advances in electronic taste-sensing technologies, particularly lipid/polymer membrane-based systems, enable quantitative evaluation of bitterness modulation. Because taste sensors primarily reflect peripheral physicochemical interactions rather than central sensory processing, this review further discusses the potential utility of the bitterness sensor BT0 for evaluating the peripheral component of bitterness suppression, drawing primarily on studies using MSG and representative umami peptides. Collectively, current evidence suggests that umami-mediated taste modulation may provide a rational framework for improving oral pharmaceutical palatability.

## 1. Introduction

Bitterness is an evolutionarily conserved sensory modality that functions as a defensive mechanism against the ingestion of potentially harmful compounds [1,2,3] and is an inherent property of many orally administered active pharmaceutical ingredients (APIs). These compounds elicit bitterness through activation of human bitter taste receptors (TAS2Rs), often resulting in reduced palatability, decreased patient adherence, and suboptimal therapeutic outcomes [4,5,6].

The clinical relevance of bitterness is particularly pronounced in pediatric and geriatric populations, where altered taste sensitivity and impaired swallowing function may further exacerbate aversion to oral medications [7,8]. Consequently, control of bitterness remains a central challenge in oral dosage form design.

Conventional taste-masking strategies, including film coating, microencapsulation, cyclodextrin inclusion complexes, and ion-exchange resins, primarily aim to reduce direct drug–receptor interactions through physicochemical barriers [9,10,11]. While effective in specific formulations, these approaches do not directly address receptor-level activation and often show limited applicability depending on the physicochemical properties of the API [12].

Against this background, increasing attention has been directed toward mechanism-based approaches that modulate taste signaling itself rather than solely preventing drug–receptor contact [13]. From an evolutionary perspective, umami functions as a sensory cue for amino acid-rich foods and contributes to nutrient recognition. This physiological role provides a biological basis for understanding interactions with other taste modalities, including modulation of bitter taste perception [13,14].

Among the basic taste modalities, umami has attracted particular interest as a modulatory system influencing not only taste enhancement but also cross-modal taste interactions, including bitterness perception [13,14]. One proposed mechanism involves direct interactions of certain umami compounds with specific human bitter taste receptors, including hTAS2R16. Rhyu et al. reported that γ-glutamyl-glutamic acid (Glu-Glu), monosodium glutamate (MSG), and L-theanine inhibit hTAS2R16-mediated bitter signaling by targeting the receptor’s probenecid-binding pocket, as demonstrated by mutagenesis analysis [14].

Although a variety of umami substances have been investigated for bitterness suppression, the most detailed mechanistic and quantitative evidence is currently available for monosodium glutamate (MSG) and representative umami peptides. Accordingly, while this review covers the broader field of umami-mediated bitterness suppression, particular emphasis is placed on these representative compounds to illustrate current advances in receptor-level mechanisms and sensor-based evaluation.

Umami compounds, primarily L-glutamate and 5′-ribonucleotides, signal through the T1R1/T1R3 heterodimeric receptor, which functions as the primary umami receptor in mammals [15]. This receptor exhibits strong synergistic activation in response to L-glutamate in combination with 5′-ribonucleotides such as inosine monophosphate (IMP) and guanosine monophosphate (GMP), resulting in enhanced signal transduction compared with glutamate alone [16]. In addition, umami stimuli have been reported to modulate bitter taste perception through peripheral receptor interactions, whereas taste mixture interactions such as bitterness inhibition by sweeteners have been described at the perceptual level of gustatory processing [17,18]. Beyond their classical role as flavor enhancers, umami substances contribute to overall taste balance and integrated flavor perception rather than acting as isolated taste stimuli [19].

Furthermore, taste perception arises from the coordinated activity of multiple receptor systems, including TAS2Rs and T1R1/T1R3, whose signals are integrated within neural circuits in the central nervous system [19,20,21]. Accordingly, current evidence suggests that bitterness suppression may involve multiple processes extending beyond receptor-level interactions to include perceptual modulation and central sensory integration [19,20,21]. This organization may contribute to cross-modal interactions among basic taste modalities [19,20,21].

In parallel, analytical technologies for objective taste evaluation have advanced significantly. To date, taste sensors based on various principles and methodologies have been reported, including potentiometric, voltammetric, and impedimetric types, and sensors using biological tissues [22,23,24,25,26]. In particular, for applications in pharmaceutical evaluation, two prominent systems are widely recognized. The Astree taste sensor has been used to evaluate overall taste profiles and bitterness reduction [27,28], while the Insent taste sensor is well known for utilizing lipid/polymer membranes designed to respond to distinct basic taste qualities. The lipid/polymer membrane-based sensors—such as the BT0 bitterness sensor—enable quantitative and reproducible assessment of peripheral bitterness and its modulation by taste-active compounds [29,30,31].

When combined with multivariate statistical methods and machine learning approaches, these systems allow quantitative modeling of taste modulation and prediction of formulation-dependent effects [32,33,34].

**Figure 1:**
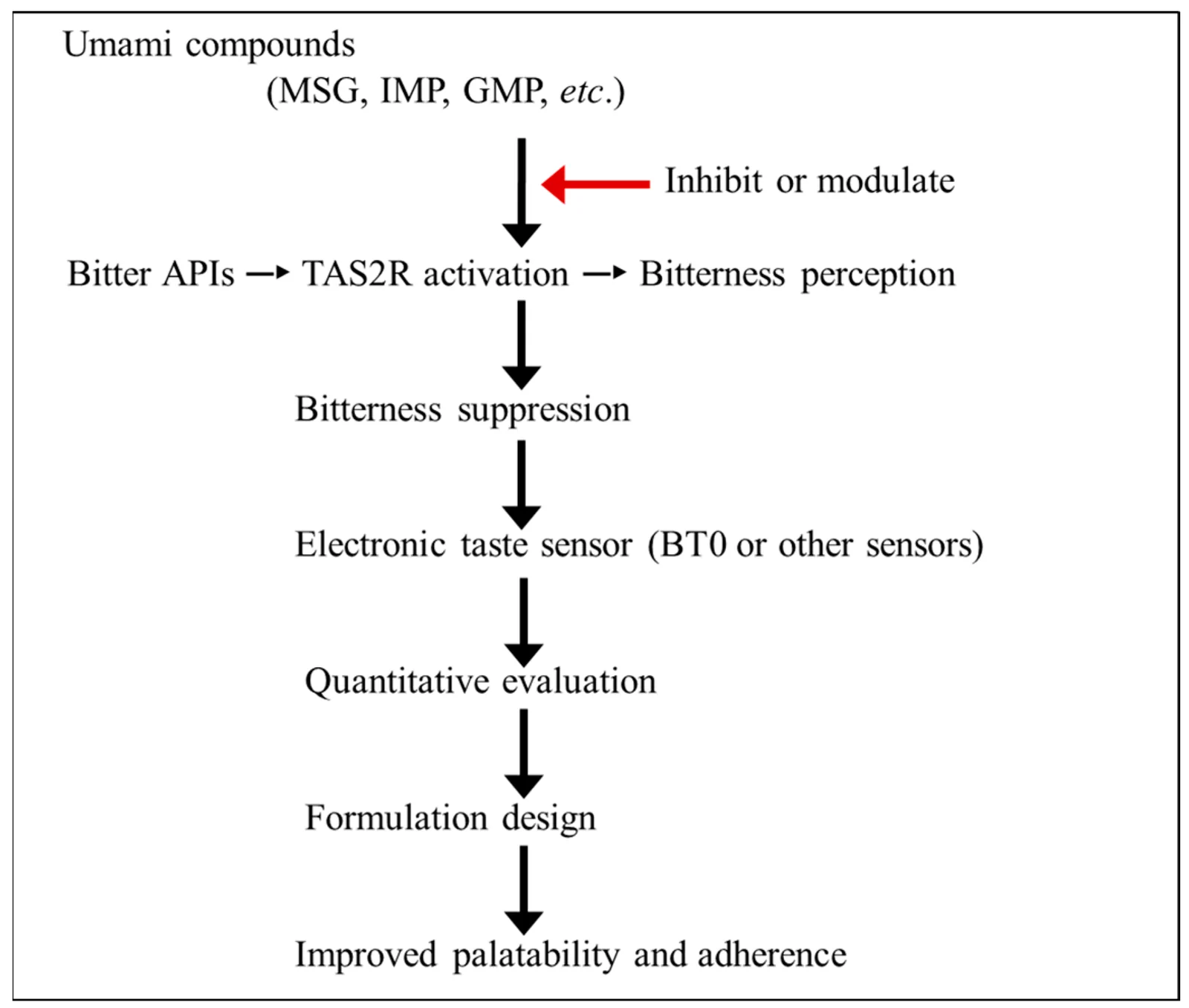
Integrated framework of umami-mediated bitterness suppression, sensor-based evaluation, and pharmaceutical formulation design.

Despite substantial progress in understanding umami-mediated bitterness suppression, the relationships among peripheral and central mechanisms, quantitative taste evaluation, and pharmaceutical formulation design have not been comprehensively integrated. Figure 1 summarizes the conceptual framework of this review, linking current knowledge of bitterness suppression with BT0 sensor-based evaluation and its potential application to pharmaceutical formulation design. Accordingly, this review provides an integrated overview of umami-mediated bitterness suppression from receptor, sensory, neural, and analytical perspectives, while recognizing that the available evidence differs in both experimental level and mechanistic certainty. Building on previous evidence demonstrating bitterness suppression by representative umami compounds, particularly MSG and umami peptides [14], this review examines whether these peripheral effects can be quantitatively evaluated using the BT0 bitterness sensor. The results of this sensor-based approach are discussed in the context of objectively assessing MSG-mediated bitterness suppression and its potential utility for pharmaceutical taste evaluation.

The literature included in this review was identified primarily through PubMed searches for articles published from 2000 to the present using combinations of keywords including umami, monosodium glutamate (MSG), bitterness suppression, taste masking, pharmaceutical, drug, electronic tongue, taste sensor, and TAS2R. Additional relevant publications were identified through manual screening of the reference lists of key articles. The selected literature was critically evaluated and synthesized to provide a comprehensive overview of the current understanding of umami-mediated bitterness suppression in pharmaceutical applications. Based on this literature search, particular emphasis is placed on MSG and representative umami peptides because they currently provide the strongest experimental evidence for bitterness suppression in pharmaceutical applications. These representative compounds are used throughout this review to illustrate current advances in mechanistic understanding, sensor-based evaluation, and pharmaceutical formulation.

## 2. Bitter Taste Mechanisms and Pharmaceutical Relevance

Bitterness perception in humans is primarily mediated by the TAS2R family of G protein-coupled receptors (GPCRs), which function as broadly tuned chemosensory detectors capable of recognizing structurally diverse bitter ligands [35,36,37]. Recent structural and computational studies have further demonstrated that TAS2Rs exhibit substantial ligand promiscuity, enabling a relatively small receptor repertoire (~25 subtypes) to detect a broad chemical space of xenobiotics, including numerous pharmacologically relevant compounds [36,38]. Although this broad tuning is generally considered an evolutionary advantage, it presents a major challenge in pharmaceutical development, where unintended taste activation is undesirable.

In drug development, bitterness is frequently associated with multiple classes of APIs, including antibiotics, alkaloids, antihistamines, and central nervous system agents [39,40,41]. These compounds often share physicochemical characteristics such as moderate lipophilicity (logP), aromatic ring structures, and hydrogen bond acceptor/donor patterns, which contribute not only to receptor–ligand interactions but also to membrane permeability and pharmacokinetic behavior. Recent quantitative structure–taste relationship (QSTR) studies suggest that bitterness cannot be attributed to a single molecular descriptor, but rather emerges from multidimensional chemical features, including electronic distribution, molecular flexibility, and topological indices [40,42].

Importantly, bitterness intensity is not determined solely by ligand–receptor binding affinity. Rather, it reflects the integration of multiple biological processes, including receptor activation kinetics, intracellular signaling amplification, receptor expression patterns, and genetic polymorphisms within TAS2R gene families [37,43,44]. Advances in genomics and functional assays have demonstrated substantial interindividual variability in TAS2R responsiveness, which may contribute to differences in bitterness perception thresholds and aversive behavioral responses. Such variability appears particularly relevant in pediatric populations, where heightened sensory sensitivity and behavioral aversion to bitterness can significantly influence medication acceptability [45].

From a pharmaceutical perspective, bitterness should therefore be considered not merely a sensory attribute, but also an important determinant of medication adherence and therapeutic outcomes. Poor palatability has been associated with dose omission, incomplete treatment courses, and reduced clinical efficacy, particularly in chronic therapies and pediatric formulations [39,45]. Although in silico prediction models based on molecular descriptors—such as lipophilicity, molecular weight, and polar surface area—have been proposed, their predictive performance remains limited. This limitation likely reflects the inherently multilevel nature of taste perception, which may involve peripheral receptor recognition, intracellular signaling dynamics, sensory integration, and higher-order neural processing [41,42].

Recent advances integrating molecular docking, machine learning, and receptor-based modeling have improved the prediction of bitterness potential during early-stage drug development [43,44,45]. However, translating these predictions into practical pharmaceutical formulation strategies remains challenging. In particular, bridging molecular-level predictions with perceptual outcomes requires a more integrated conceptual framework linking chemical structure, receptor activation profiles, sensory integration processes, and sensor-based evaluation. Such an approach may facilitate the rational design of more palatable pharmaceutical formulations while promoting closer integration between molecular pharmacology, sensory science, and pharmaceutical technology. Among emerging mechanism-based approaches, umami-mediated taste modulation has received increasing attention because it may attenuate bitterness through multiple levels of taste processing rather than solely through physicochemical masking. The following section summarizes current evidence regarding representative umami compounds and their potential roles in bitterness suppression.

## 3. Current and Emerging Strategies for Bitterness Suppression in Pharmaceuticals

**Figure 2:**
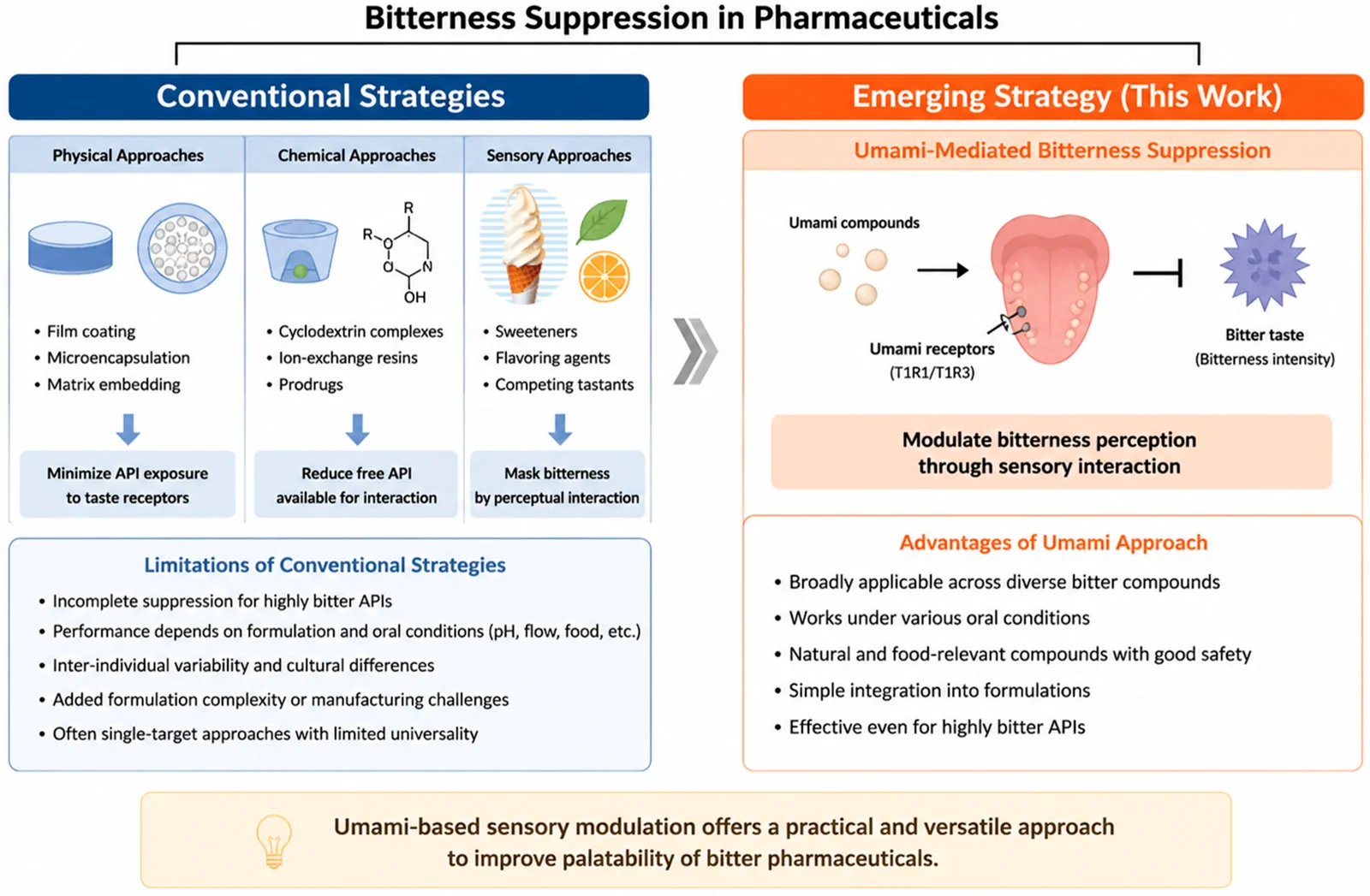
Emerging strategies for bitterness suppression in pharmaceuticals.

Figure 2 provides an overview of current and emerging strategies for bitterness suppression in pharmaceutical development. Bitterness suppression strategies in pharmaceutical development can generally be classified into physical, chemical, sensory, receptor-oriented, and data-driven approaches, each addressing different aspects of drug-related bitterness as shown in Figure 2 (left) [46,47]. However, because bitterness perception depends on both API properties and oral physiological conditions, no single strategy has achieved universal applicability across structurally diverse compounds.

Physical approaches primarily aim to minimize direct exposure of APIs to taste receptors. Representative techniques include film coating, microencapsulation, and matrix embedding. Polymer-based systems, particularly methacrylate copolymers such as Eudragit, form diffusion barriers that delay drug release in the oral cavity. These approaches are widely applicable and relatively easy to implement in formulation design; however, their effectiveness may be compromised by incomplete coating, mechanical instability, and variability in dissolution behavior under physiological conditions [47,48]. Although recent advances in micro- and nano-encapsulation technologies have improved release control and coating uniformity, challenges related to scalability and manufacturing reproducibility remain significant.

Chemical approaches reduce bitterness by modifying the molecular environment surrounding APIs. Common strategies include cyclodextrin inclusion complexes, ion-exchange resins, and prodrug design [49,50]. Cyclodextrins suppress bitterness by encapsulating hydrophobic regions of APIs, thereby decreasing the concentration of free drug available for receptor interaction. However, dissociation in saliva may still permit partial receptor activation. Similarly, ion-exchange systems are influenced by pH and ionic strength, limiting formulation robustness under variable oral conditions. Prodrug strategies offer mechanistic advantages because they can temporarily mask bitter functional groups while preserving systemic drug activity after bioconversion, although their practical application remains limited.

Sensory approaches focus on altering perceptual responses rather than preventing receptor exposure. Sweeteners, flavoring agents, and competing tastants can reduce perceived bitterness through taste mixture interactions [51,52,53]. These methods are simple and formulation-compatible, but their effects are often insufficient for highly bitter APIs and may vary substantially among individuals. In addition, age, cultural background, and sensory sensitivity strongly influence overall palatability. Recent studies further indicate that aroma–taste interactions and trigeminal stimulation contribute significantly to bitterness perception and masking efficiency. Receptor-oriented approaches targeting TAS2R-mediated signaling have attracted increasing interest as potentially selective suppression strategies. These include competitive antagonists, allosteric modulators, and pathway-specific inhibitors designed to interfere directly with bitter taste receptor activation [54]. While advances in structural biology and high-throughput screening have accelerated identification of candidate compounds, clinical translation remains challenging because of safety concerns, receptor subtype selectivity, and regulatory complexity.

Electronic taste-sensing systems (electronic tongues), particularly lipid/polymer membrane-based sensors capable of peripheral quantitative taste evaluation, provide an objective and highly reproducible platform by converting chemical interactions into electrical signal patterns [55]. In parallel, computational ligand discovery and predictive modeling approaches have accelerated identification of candidate bitterness-modulating compounds [56]. In addition, Quality by Design (QbD)-based optimization and emerging in vitro–in vivo correlation (IVIVC) frameworks may improve translation of analytical measurements into clinically relevant sensory outcomes. Overall, each bitterness suppression strategy possesses distinct advantages and limitations, and its effectiveness varies depending on API characteristics and formulation conditions. Therefore, future pharmaceutical design will likely require flexible combinations of physicochemical, sensory, receptor-oriented, and predictive analytical approaches to achieve reliable and broadly applicable bitterness suppression. Among emerging sensory modulation strategies, increasing attention has recently been directed toward umami-mediated bitterness suppression because accumulating evidence suggests that it may modulate bitterness perception through multiple peripheral and sensory mechanisms, as summarized in Figure 2 (right). The mechanistic basis of the umami taste system is discussed in the following section.

## 4. Umami Taste System

Umami is recognized as a fundamental taste modality that signals the presence of amino acid-rich nutrients and plays important roles in dietary selection and metabolic regulation. It is primarily elicited by L-glutamate and potentiated by 5′-ribonucleotides such as IMP and GMP, which exhibit pronounced synergistic enhancement of taste intensity [57,58]. This synergistic interaction is a defining feature of the umami system and contributes to its high sensitivity and functional specificity in nutrient detection.

At the molecular level, umami perception is mediated by the heterodimeric T1R1/T1R3 receptor, a class C G protein-coupled receptor expressed in taste receptor cells. Structural and functional studies have demonstrated that L-glutamate binds to the Venus flytrap domain of T1R1, inducing conformational changes that are further stabilized by nucleotides acting as positive allosteric modulators [59,60,61]. This cooperative binding mechanism enhances receptor activation efficiency and distinguishes umami signaling from many other taste modalities that rely predominantly on orthosteric ligand binding. Recent advances in receptor modeling and mutagenesis studies have further clarified ligand-binding dynamics and subtype-specific sensitivity, providing a molecular basis for interindividual variability in umami perception. Upon receptor activation, intracellular signaling cascades are initiated through G protein coupling, leading to the activation of phospholipase C β2 (PLCβ2), production of inositol 1,4,5-trisphosphate (IP3), and subsequent release of intracellular calcium. These events trigger depolarization of taste receptor cells and neurotransmitter release to afferent gustatory neurons [62]. The amplification properties of this signaling cascade contribute to strong perceptual responses to umami stimuli even at relatively low concentrations and may partly underlie modulatory effects on other taste modalities.

Beyond the oral cavity, T1R1/T1R3 receptors are expressed in various extraoral tissues, including the gastrointestinal tract, pancreas, and brain, where they function as nutrient sensors involved in digestion, hormone secretion, and metabolic regulation [63,64]. This broader physiological distribution suggests that umami signaling is integrated into systemic nutrient-sensing networks, extending its functional relevance beyond taste perception alone.

Importantly, umami is strongly associated with positive hedonic responses and activation of reward-related neural circuits, including the insular cortex and orbitofrontal cortex. These observations further support the view that umami functions not only as an independent taste modality but also as a modulator of sensory perception through receptor-mediated signaling and interactions with other taste qualities.

Recent evidence suggests that umami functions not only as an independent taste modality but also as a modulator of sensory perception through receptor-mediated signaling mechanisms and interactions with other taste qualities [64,65]. This modulatory role is particularly relevant in the context of taste interactions, where umami has been reported to attenuate aversive sensations such as bitterness. Such effects may involve peripheral taste interactions and partially overlapping intracellular signaling pathways.

This perspective positions the umami system as a functional interface linking nutrient detection, sensory integration, and hedonic evaluation. In this context, accumulating sensory, neurophysiological, and receptor-level evidence suggests that the involvement of umami in bitterness suppression may involve coordinated sensory and signaling processes rather than a simple receptor-level interaction. Consequently, the umami system may provide a promising mechanistic foundation for the rational design of palatable pharmaceutical formulations, particularly when combined with sensor-based evaluation and quantitative analytical approaches. The following section discusses how these molecular and sensory characteristics may contribute to multilevel mechanisms of umami-mediated bitterness suppression.

## 5. Mechanisms of Umami-Mediated Bitterness Suppression

**Figure 3:**
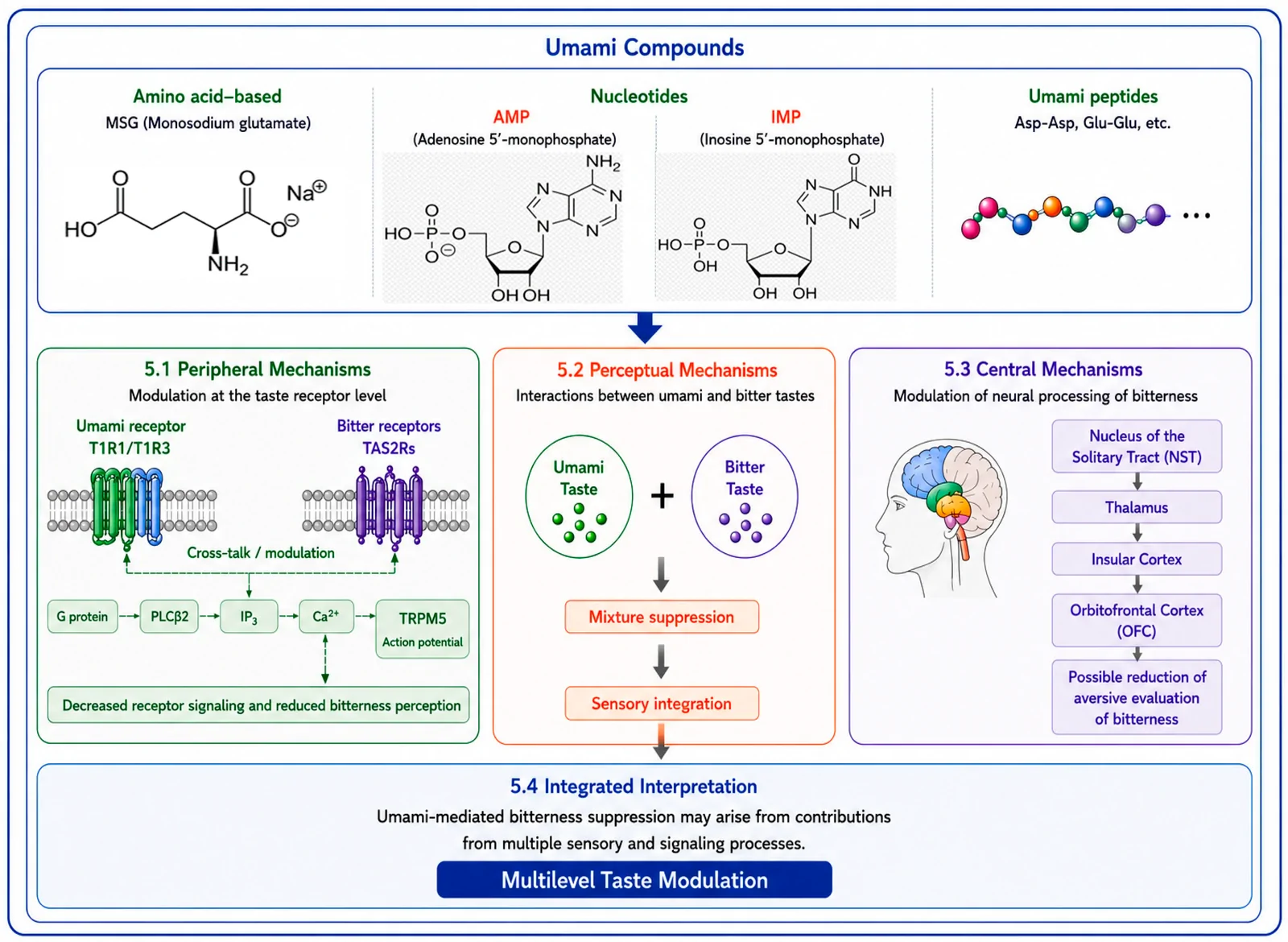
Conceptual framework of multilevel mechanisms underlying umami-mediated bitterness suppression. This figure summarizes the conceptual framework presented in Section 5. Section 5.1 illustrates the proposed peripheral receptor-associated mechanisms involving T1R1/T1R3- and TAS2R-related signaling. Section 5.2 summarizes perceptual and mixture interactions that may contribute to bitterness attenuation. Section 5.3 depicts the potential involvement of central gustatory processing in modulating bitterness perception. Section 5.4 integrates these findings into a multilevel taste modulation model, emphasizing that the relative contribution of each process remains to be fully elucidated.

Current evidence suggests that umami-mediated bitterness suppression may involve multiple stages of taste processing, including peripheral receptor responses, perceptual taste interactions, and central sensory integration processes [66,67]. Although findings at each level provide valuable insights, the relationships among peripheral receptor events, perceptual interactions, central neural processing, and sensor-based findings remain largely inferential rather than being supported by direct experimental evidence. Unlike conventional bitterness-masking strategies, which primarily reduce direct interactions between APIs and taste receptors through physicochemical or formulation-based approaches, umami-mediated suppression may involve modulation of multiple stages of taste processing. Accordingly, Figure 3 summarizes the conceptual framework discussed in this section and should be regarded as a conceptual model summarizing current evidence rather than an established mechanistic pathway.

### 5.1. Peripheral Mechanisms

At the peripheral level, bitterness is primarily detected by TAS2R receptors, whereas umami perception is mediated by the heterodimeric T1R1/T1R3 receptor complex [68,69,70]. Although these receptor systems belong to distinct GPCR families, several studies have suggested partial overlap within downstream intracellular signaling pathways.

Both TAS2R- and T1R-mediated pathways have been reported to share several intracellular signaling components, including G protein activation, PLCβ2, IP3 production, and intracellular calcium mobilization [71,72]. However, the extent to which these shared signaling components directly contribute to bitterness suppression remains unclear.

In addition, L-glutamate and certain umami-active peptides have been reported to attenuate bitterness-related responses under specific experimental conditions [73]. Nevertheless, it remains uncertain whether these effects arise from direct receptor-level antagonism or from modulation occurring at downstream signaling or perceptual processing stages.

Furthermore, the magnitude of these effects appears to depend on factors such as ligand concentration ratios, temporal presentation patterns, and local oral environmental conditions, suggesting that umami-mediated bitterness suppression may represent a context-dependent sensory modulation phenomenon rather than a fixed receptor interaction mechanism.

### 5.2. Perceptual and Mixture Mechanisms

At the perceptual level, interactions between umami and bitter stimuli may involve mixture suppression phenomena, in which simultaneously presented taste qualities modify each other’s perceived intensity [74,75].

Co-administration of umami compounds and bitter substances has been reported, under certain conditions, to reduce perceived bitterness intensity. However, these effects do not appear to be uniform and may depend on factors such as concentration ratios, timing of presentation, and formulation conditions.

In addition, pharmaceutical and oral-environmental factors—including dissolution kinetics, salivary dilution, oral residence time, and temporal release behavior—may influence effective receptor exposure and subsequent sensory integration processes.

Collectively, the available evidence suggests that umami-mediated bitterness reduction cannot be explained solely by simple additive effects, and that perceptual integration within the gustatory system may also contribute to the observed modulation. Furthermore, similar effects have been reported for structurally diverse APIs, raising the possibility that this phenomenon reflects a broader perceptual modulation mechanism rather than compound-specific physicochemical masking [76].

### 5.3. Central Neural Mechanisms

Gustatory information is transmitted from the nucleus of the solitary tract through the thalamus to higher-order gustatory regions, including the insular cortex and orbitofrontal cortex [77]. These distributed neural networks are thought to contribute not only to sensory intensity coding but also to integrated affective evaluation of taste stimuli.

Umami stimulation has been associated with the activation of reward-related neural regions, raising the possibility that such neural activity may contribute to the attenuation of the aversive responses associated with bitterness [78].

In addition, several neuroimaging studies have reported alterations in functional connectivity within gustatory and reward-related brain regions during simultaneous umami–bitter stimulation [79]. These observations suggest that umami stimuli may influence not only bitterness intensity perception but also the affective evaluation of taste stimuli. However, evidence directly linking these central neural processes to pharmaceutical bitterness suppression remains limited and is largely inferred from broader studies of taste perception and hedonic evaluation. Therefore, the mechanisms underlying these central neural interactions require further investigation.

### 5.4. Integrated Interpretation of Umami-Mediated Bitterness Suppression

Taken together, current evidence suggests that umami-mediated bitterness reduction may arise from contributions from multiple sensory and signaling processes, including peripheral receptor responses, perceptual mixture effects, and central neural processing.

These effects are also likely influenced by substantial interindividual variability, including TAS2R genetic polymorphisms, prior sensory experience, age-related differences in taste responsiveness, and contextual sensory conditions [80,81]. The involvement of these multiple factors may partly explain why bitterness suppression effects are often context-dependent and variable among individuals. In contrast, simplified models based solely on receptor antagonism or physicochemical shielding may not fully account for such variability. Accordingly, umami-mediated bitterness reduction may be more appropriately interpreted as a multilevel taste modulation phenomenon involving coordinated sensory, signaling, perceptual, and neural processes rather than a single mechanistic pathway [82]. This perspective may provide a useful conceptual basis for future pharmaceutical palatability research and for the development of umami-based formulation strategies integrated with sensor-based evaluation systems and quantitative analytical approaches.

## 6. Human and Sensor-Based Evidence for Umami-Induced Bitterness Suppression

The literature demonstrating the bitter-masking effect of umami substances on pharmaceuticals is limited.

Several independent studies have reported bitterness suppression by umami compounds using both sensory and analytical approaches.

Human sensory studies have provided evidence that umami substances can attenuate bitterness perception. Mennella et al. demonstrated that MSG significantly reduces the bitterness of quinine in both pediatric and adult populations, indicating a broad suppressive effect across age groups [83]. In a large-scale human sensory study involving more than 100 participants, MSG also significantly reduced the perceived bitterness of urea, denatonium benzoate, and quinine, further supporting its broad bitterness-suppressive properties [83].

Although human sensory evaluation is widely regarded as the standard method for assessing taste perception in pharmaceutical formulations, it has inherent limitations, including subjectivity, interindividual variability, and experimental constraints [84]. These limitations become particularly important when evaluating subtle taste-modifying effects such as umami-induced bitterness suppression, where high reproducibility and quantitative resolution are required. Accordingly, complementary analytical approaches are desirable.

Electronic taste-sensing systems (electronic tongues) provide an objective and highly reproducible platform by converting chemical interactions into electrical signal patterns [85,86,87]. Unlike receptor-specific biological assays, these systems integrate multiple physicochemical interactions at the sensor membrane interface, including electrostatic interactions, hydrophobic adsorption, and competitive molecular interactions, thereby partially mimicking early-stage taste perception processes.

Previous studies have demonstrated the usefulness of electronic tongue systems for evaluating pharmaceutical bitterness and taste-masking effects. Various platforms, including pattern-recognition-based electronic tongues and lipid/polymer membrane-based taste sensors, have been applied to characterize bitterness profiles of active pharmaceutical ingredients (APIs) and to assess the effectiveness of taste-masking strategies.

For example, the Astree taste sensor system has been used to evaluate overall taste profiles and bitterness reduction after formulation with masking agents through multivariate pattern recognition approaches [27,28]. Specifically, recent studies utilized principal component analysis (PCA) on sensor signals to quantify bitterness distance (D) for assessing the bitterness of active pharmaceutical ingredients and predicting human sensory responses [27]. Furthermore, this system has been successfully applied to evaluate the taste-masking efficiency of various excipients in pediatric liquid formulations, demonstrating its ability to objectively discriminate complex taste profiles and monitor palatability improvements achieved by masking agents [28].

In contrast, lipid/polymer membrane-based taste sensors, such as the Insent taste sensor system, enable quantitative evaluation of individual taste qualities by detecting specific physicochemical interactions at the sensor membrane interface [29,57]. By utilizing task-specific lipid membranes, this system can independently quantify initial taste responses and changes in membrane potential caused by adsorption (CPA), which corresponds to bitterness persistence and is expressed in millivolts (mV). These measurements have been reported to correlate well with human sensory scores [31,57].

In this review, bitterness was quantitatively evaluated using the Insent taste sensor system equipped with bitterness-specific sensor membranes. While electronic tongues such as the Astree taste sensor are primarily designed for overall taste profiling based on pattern recognition, the Insent taste sensor utilizes lipid/polymer membrane technology designed to quantify individual taste attributes [57], thereby facilitating objective evaluation of bitterness suppression achieved by additive substances. Although the Astree taste sensor is applied to assess bitterness reduction mediated by sweeteners and general taste-masking excipients in pharmaceuticals, to the authors’ knowledge, there are no reports evaluating pharmaceutical bitterness suppression achieved by umami substances like MSG.

Despite these advances in analytical approaches for taste evaluation, quantitative assessment of bitterness suppression mediated by umami compounds, particularly in pharmaceutical formulations, remains insufficiently explored. Therefore, lipid/polymer membrane-based taste sensors currently represent one of the few quantitative approaches available for evaluating the peripheral effects of umami compounds on pharmaceutical bitterness.

The authors have previously conducted a series of studies using AN0-, AC0-, and BT0-type lipid/polymer membrane electrodes for the evaluation of pharmaceutical bitterness and bitterness suppression. The detecting part of the taste-sensing system consists of a reference electrode and a taste sensor that acts as the working electrode and is composed of various lipid/polymer membranes with different physicochemical properties and response characteristics [31]. The lipid/polymer membranes used as bitterness sensors in these studies were AN0 (phosphoric acid di-n-decyl ester/dioctyl phenylphosphonate), AC0 (hexadecanoic acid/dioctyl phenylphosphonate), and BT0 (phosphoric acid di-n-decyl ester/bis(1-butylpentyl) adipate, tributyl O-acetylcitrate). Although all three membranes are responsive to basic bitter substances, differences in membrane composition result in distinct response profiles.

Academic literature evaluating the effects of umami substances on pharmaceuticals using taste sensors remains scarce, with most of these studies originating from the authors’ research group. Here, a representative work by the authors in this area is summarized.

As discussed later, BT0 has attracted particular interest because its response characteristics may partially reflect receptor-related properties of certain bitter compounds.

**Figure 4:**
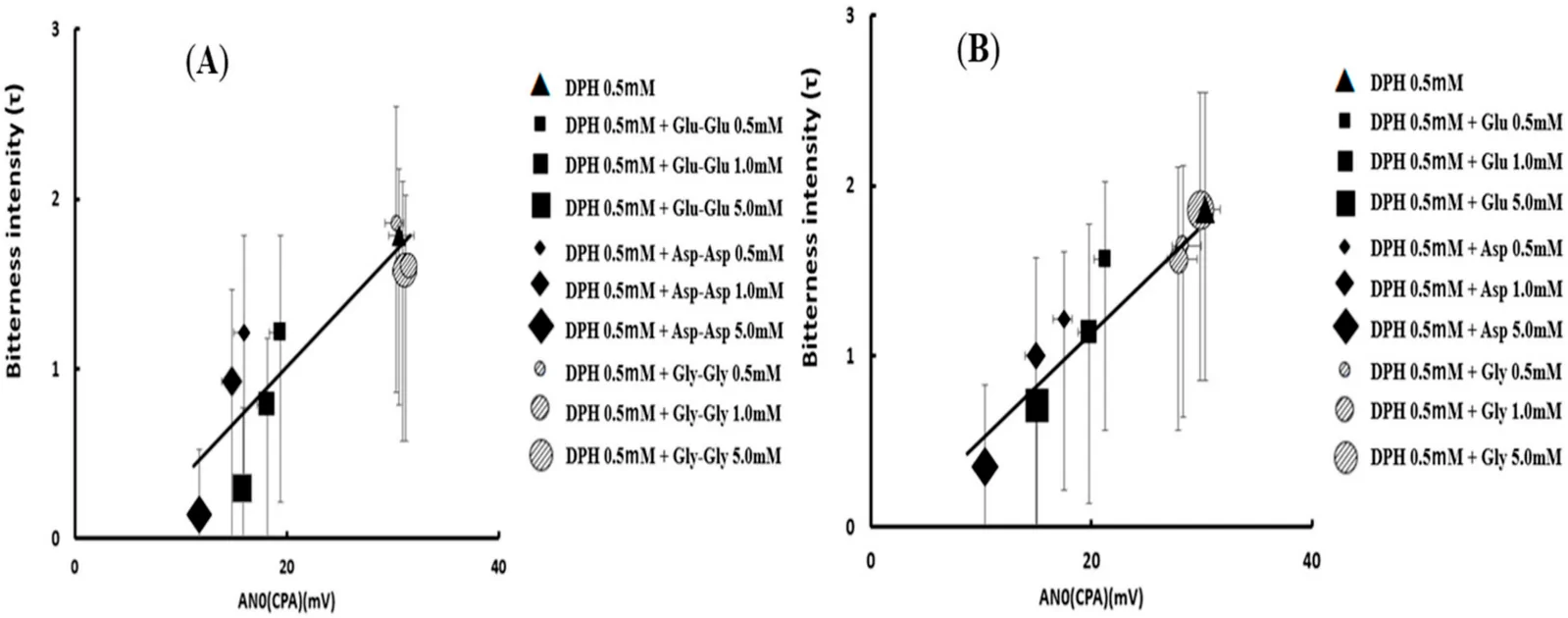
Correlation between taste sensor output AN0 (CPA) and bitterness intensity by gustatory sensation test (reproduced from [88]). (**A**) DPH solutions containing three dipeptides at different concentrations (n = 10, rs = 0.817, *p* < 0.01). (**B**) DPH solutions containing three amino acids at different concentrations (n=10, $r_{s}=0.970$, p<0.001). Statistical significance was evaluated by Spearman’s correlation test.

Okuno et al. demonstrated that the umami dipeptides Glu-Glu and Aspartyl-aspartic acid (Asp-Asp), as well as their constituent amino acids, significantly reduced the bitterness of diphenhydramine hydrochloride (DPH) in both human sensory tests and taste sensor measurements, as shown in Figure 4. CPA described in the figure caption is an index of aftertaste intensity that reflects the adsorption of taste compounds onto the lipid/polymer membrane of the taste sensor. The observed correlation between sensor responses and sensory scores, together with receptor-binding and molecular docking studies, suggested that interactions with hTAS2R14 may contribute to the observed bitterness suppression [88].

**Figure 5:**
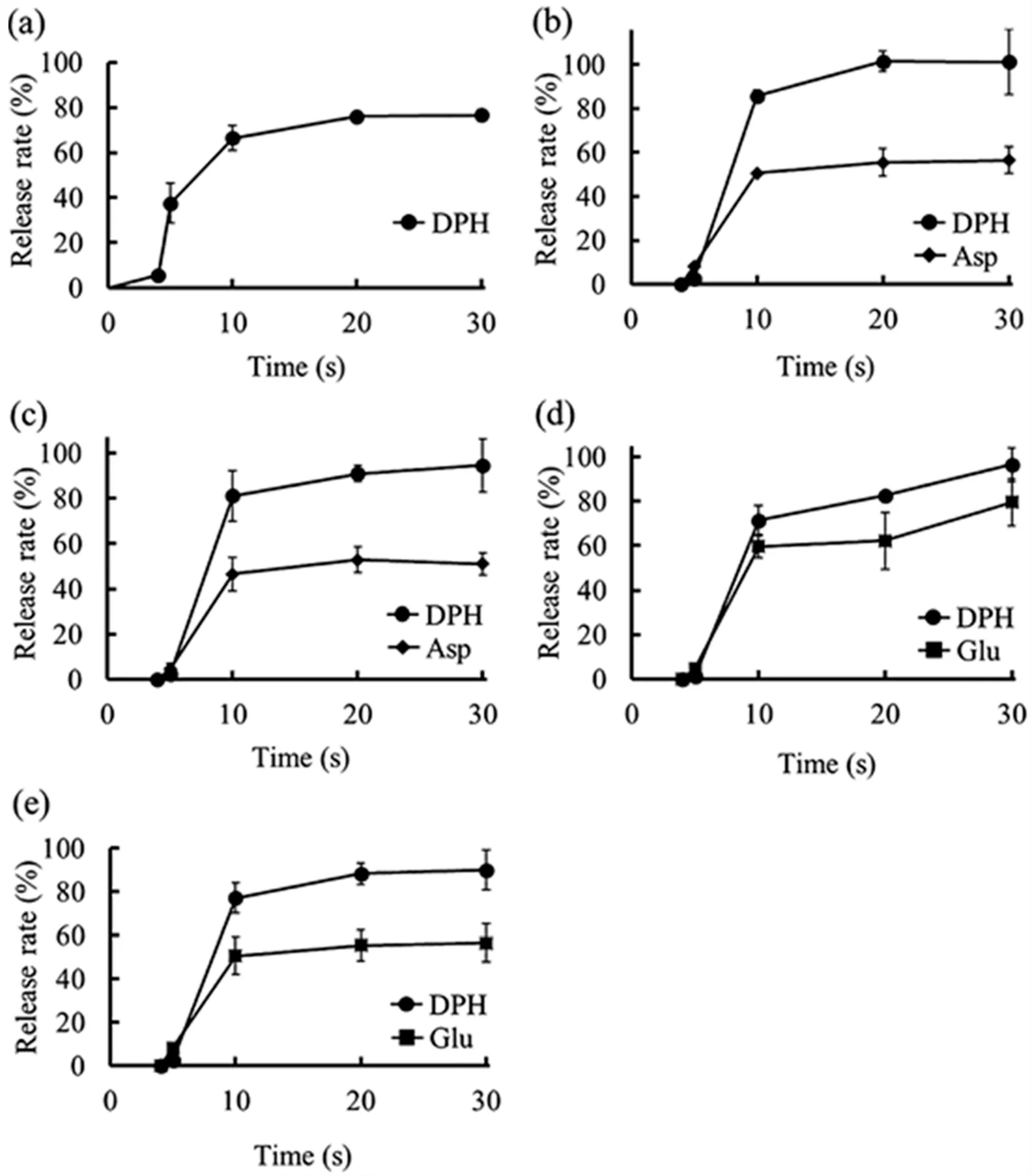
DPH and Asp/Glu release (%) in the dissolution medium over time as measured by the OD-mate (reproduced from [89]); (**a**–**e**) correspond to OFDMTs ((A)–(E)), respectively. Data are expressed as mean ± SD (n = 3). For the formulations of the five OFDMTs, see Table 1.

**Figure 6:**
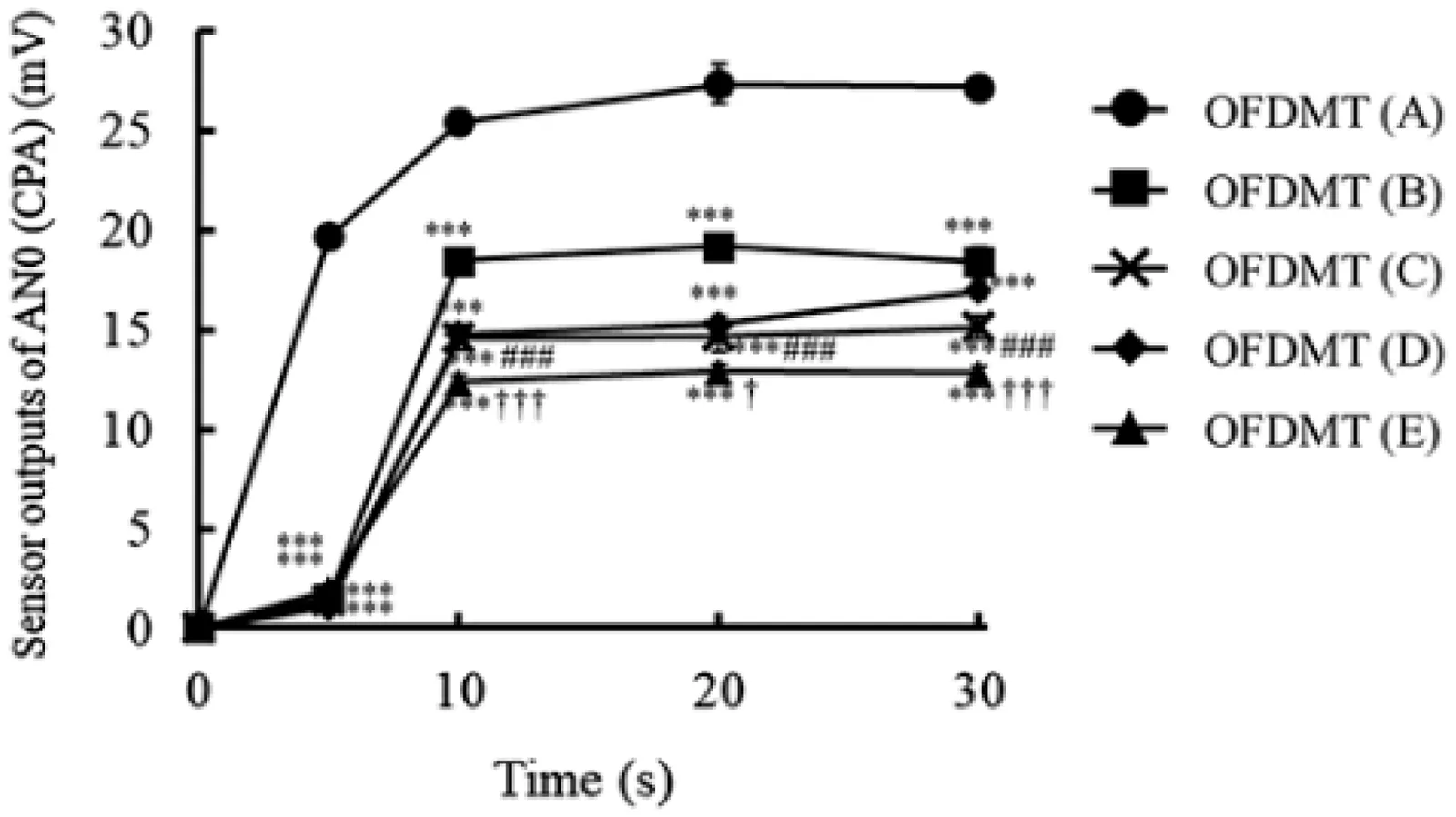
AN0 (CPA) taste sensor output–time profiles for five OFDMT formulations obtained in the OD-mate dissolution test; data are expressed as mean ± SD (n = 3). *** *p* < 0.001 vs. (A), ### *p* < 0.001 vs. (B), † *p* < 0.05, ††† *p* < 0.001 vs. (D) (Tukey’s test) (reproduced from [89]).

Building on these findings, orally fast-disintegrating mini-tablets (OFDMTs) containing DPH and the umami amino acids L-aspartic acid (Asp) or L-glutamic acid (Glu) were subsequently developed as a practical dosage form for bitterness masking [89]. The formulations were prepared by direct compression and exhibited comparable physical characteristics, including tablet size, weight, and hardness. Evaluation using an oral cavity-mimicking disintegration system demonstrated that all formulations rapidly disintegrated within 10 s. Furthermore, a simplified dissolution test designed to simulate conditions in the oral cavity was conducted over 30 s, confirming the rapid release of both DPH and the incorporated amino acids as shown in Figure 5. Taste sensor measurements of samples collected during the dissolution process showed significantly lower bitterness responses for formulations containing Asp or Glu than for the control formulation without these amino acids, as shown in Figure 6. These findings indicate that incorporation of umami amino acids into DPH-loaded OFDMTs can effectively suppress bitterness during the initial period after administration while preserving the rapid disintegration and drug-release properties required for orally disintegrating dosage forms. This approach provides a practical strategy for improving the palatability and patient acceptability of bitter-tasting medicines [89].

**Table 1:** Five formulations of DPH-loaded OFDMTs (diameter: 4 mm) (reproduced from [89]). ODIFUL^®^: Granulated mixture of mannitol, crystalline cellulose, low-substituted hydroxypropyl cellulose, and POVACOAT Type MP (polyvinyl alcohol/acrylic acid/methyl methacrylate copolymer).

	DPH	Asp	Glu	ODIFUL^®^	St-Mg
OFDMT (A)	3 mg	-	-	28.68 mg	0.32 mg
OFDMT (B)	3 mg	1.37 mg	-	27.31 mg	0.32 mg
OFDMT (C)	3 mg	2.74 mg	-	25.94 mg	0.32 mg
OFDMT (D)	3 mg	-	1.51 mg	27.17 mg	0.32 mg
OFDMT (E)	3 mg	-	3.02 mg	25.65 mg	0.32 mg

**Figure 7:**
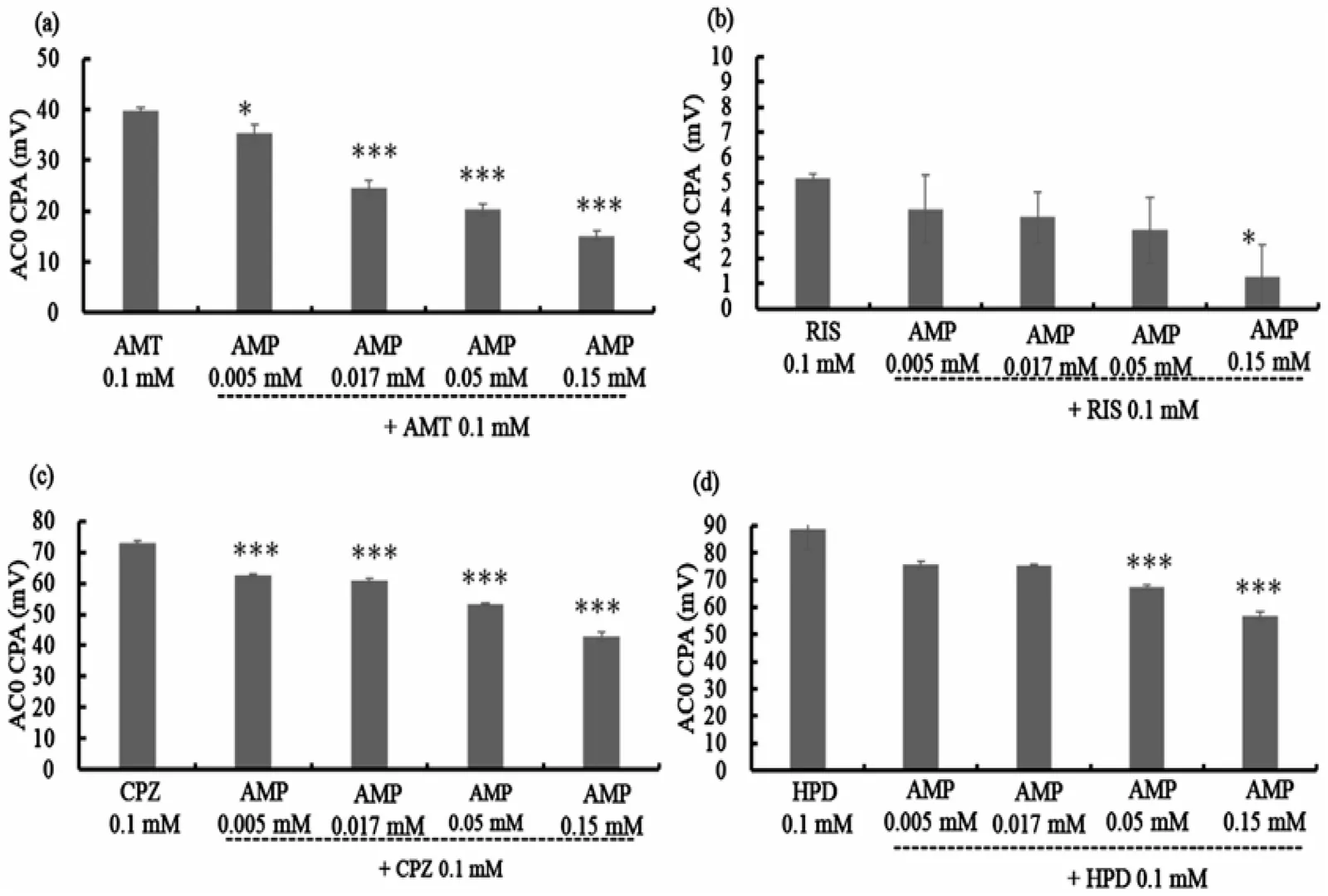
Influence of the nucleic acid-based umami compound AMP on the taste sensor output (AC0 CPA, mV) of neuropsychiatric agents: (**a**) AMT, (**b**) RIS, (**c**) CPZ, and (**d**) HPD (reproduced from [90]). Values are expressed as mean ± SD (n = 3). * *p* < 0.05, *** *p* < 0.001 vs. each drug alone (0.1 mM) (Tukey’s test).

Kawahara et al. investigated the bitterness-suppressing effect of adenosine monophosphate (AMP), an umami-related compound found in breast milk, using a taste sensor system. AMP significantly reduced the sensor responses to four highly bitter psychotropic drugs—amitriptyline (AMT), risperidone (RIS), chlorpromazine (CPZ) and haloperidol (HPD)—in a concentration-dependent manner (Figure 7) [90]. In addition, this effect was observed for the antibiotic combination sulfamethoxazole/trimethoprim, which is known for poor palatability [91]. Furthermore, molecular interactions between AMP and these drugs were suggested by MarvinSketch simulations and NMR analyses, indicating a possible physicochemical contribution to bitterness suppression [91].

**Figure 8:**
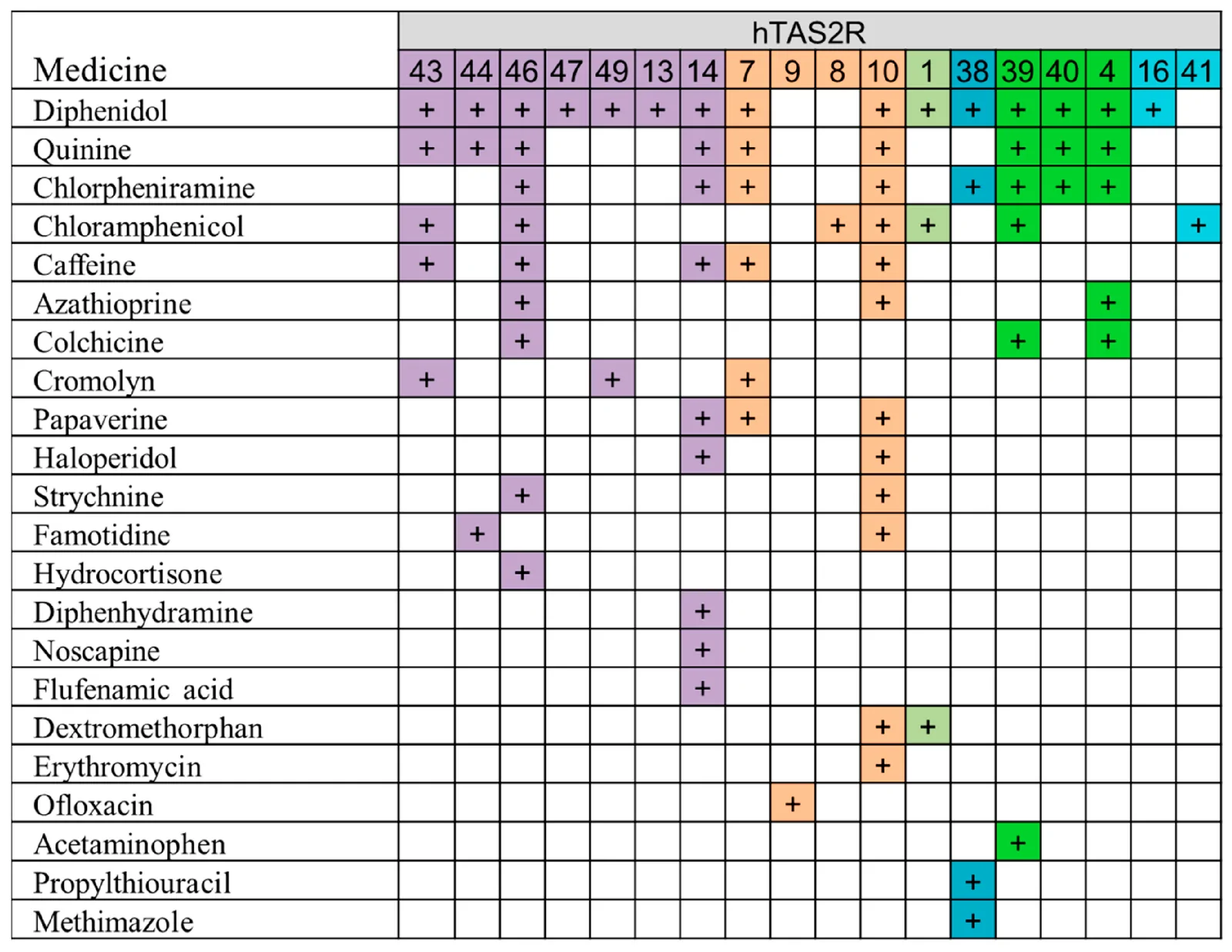
Response profiles of hTAS2Rs stimulated with 22 compounds selected from BitterDB (reproduced from [92]).

**Figure 9:**
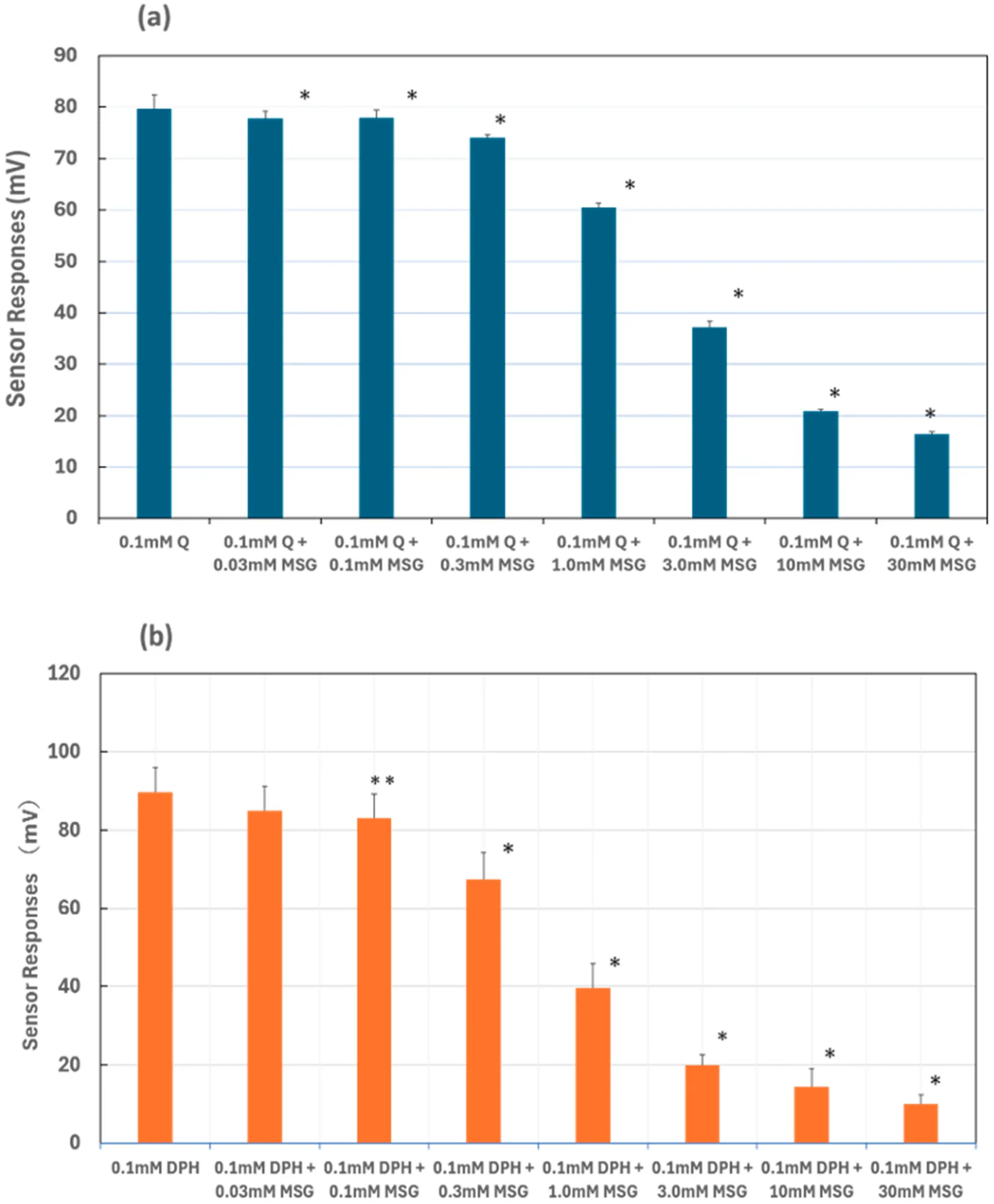
Effect of monosodium glutamate (MSG; 0–30 mM) on BT0 sensor responses to (**a**) quinine hydrochloride (Q) and (**b**) diphenhydramine hydrochloride (DPH). Values are expressed as mean ± SD (n = 12 for Q; n = 9 for DPH). Comparisons were performed against 0.1 mM Q (* *p* < 0.001 for all concentrations) and 0.1 mM DPH (** *p* < 0.05 for the initial concentration and * *p* < 0.001 for all subsequent concentrations; Tukey’s test).

In the authors’ recent study, MSG was evaluated as a bitter-masking agent using the BT0 sensor, which has been reported to respond preferentially to ligands associated with the human bitter taste receptor hTAS2R14 [92]. Bitter compounds including quinine hydrochloride and DPH, both hTAS2R14 agonists as shown in Figure 8, and propylthiouracil as an hTAS2R38 agonist as shown in Figure 8, were examined. MSG significantly suppressed BT0 sensor responses for quinine and diphenhydramine, as shown in Figure 9. However, a very low BT0 sensor response was observed for propylthiouracil (0.1, 0.5, 10 mM), a well-established agonist of hTAS2R38, as shown in Supplementary Figure S1. These findings demonstrate that electronic taste-sensing systems have the potential to evaluate bitterness suppression mediated by modifiers like MSG at the peripheral receptor level. Specifically, the BT0 sensor may preferentially reflect interactions associated with hTAS2R14-related bitter compounds, consistent with previously reported receptor-response profiles. However, these sensor responses represent peripheral physicochemical measurements and should not be interpreted as direct evidence of receptor activation or downstream neural mechanisms.

Although several mechanisms have been proposed, it remains unclear whether umami compounds suppress bitterness primarily through direct interactions with TAS2Rs or indirectly through activation of T1R1/T1R3-mediated signaling pathways. Current evidence suggests that both receptor-level interactions and downstream sensory modulation may contribute to bitterness suppression, consistent with the conceptual framework summarized in Figure 3 [93,94,95,96,97,98]. Bitter taste is primarily mediated by TAS2R receptors [93,94], whereas umami taste is detected by the heterodimeric TAS1R1/TAS1R3 receptor complex [97,98]. Although these receptor families are distinct, accumulating evidence suggests possible functional convergence and cross-modal interaction at the level of intracellular signaling within taste receptor cells [94,95,96]. Both bitter and umami signaling pathways involve partially shared downstream signaling components, including PLCβ2, IP3-mediated Ca^2+^ release, and the TRPM5 channel, which collectively regulate neurotransmitter release to afferent gustatory nerves [94,95,96]. Consequently, activation of umami receptors may alter intracellular excitability and calcium dynamics, thereby indirectly modulating cellular responses to bitter stimuli rather than acting solely through competitive receptor binding [95,97].

In addition, peripheral modulation may occur through paracrine interactions within taste buds, where ATP-mediated signaling and inhibitory feedback between taste receptor cell subtypes contribute to signal integration and sensory processing [94,98]. Type II and type III taste cells form local signaling networks mediated by ATP release and purinergic receptor signaling, enabling cross-modulation among taste modalities and influencing the overall output of gustatory afferent signaling [94,98].

Furthermore, several studies suggest that umami substances can attenuate bitterness perception without direct receptor competition, implying that suppression may occur at post-receptor or network-processing levels rather than exclusively at the initial ligand-binding step [86,95,97]. These findings are consistent with the hypothesis that umami-induced bitterness suppression may involve multilevel sensory modulation processes rather than a single-site antagonistic mechanism.

Taken together, these findings suggest that the BT0 sensor may serve as a useful tool for evaluating the peripheral component of umami-mediated bitterness suppression. Although the sensor does not directly measure receptor activation or downstream neural processing, it provides quantitative information on peripheral physicochemical responses that can be compared with human sensory findings.

Human bitterness perception likely reflects the combined influence of receptor activation, intracellular signaling, local taste-bud interactions, and higher-order sensory processing [86,95,97]. Therefore, agreement between human sensory testing and taste sensor measurements is particularly valuable when evaluating the bitterness-suppressing effects of umami compounds.

Electronic taste-sensing systems nevertheless provide a powerful platform for the objective and quantitative evaluation of umami-induced bitterness suppression. When interpreted together with human sensory and mechanistic studies, these systems may provide a translational framework linking physicochemical measurements with perceptual outcomes.

Although the currently available findings are encouraging, the overall evidence remains limited. In particular, key correlation analyses reported in the literature are based on relatively small sample sizes (e.g., n = 10), which limits the statistical power and generalizability of these findings. Furthermore, most pharmaceutical studies investigating umami-mediated bitterness suppression have been conducted by a limited number of research groups using a relatively narrow range of active pharmaceutical ingredients (APIs). Therefore, independent validation by other laboratories, together with evaluation using structurally diverse APIs, will be essential for establishing the broader applicability of these findings.

## 7. Toward an Integrated Understanding of Umami-Mediated Bitterness Suppression

The mechanistic framework of umami-mediated bitterness suppression has been discussed in Section 5, and supporting evidence from human sensory and sensor-based studies has been presented in Section 6. Here, emphasis is placed on how these findings may contribute to quantitative evaluation systems and pharmaceutical formulation design.

Understanding umami-mediated bitterness suppression requires integration of evidence from mechanistic studies, quantitative evaluation technologies, and pharmaceutical formulation research [99,100,101,102]. As discussed in the previous section, diverse umami compounds—including amino acids, nucleotides, and related substances—have been shown to attenuate bitterness across multiple pharmaceutical systems, and these effects were consistently captured by electronic taste sensors [89,90,91,92].

Although these findings collectively support the potential utility of umami compounds in pharmaceutical applications, the relationships among mechanistic observations, sensor-based measurements, and human sensory outcomes remain incompletely established and should be interpreted as complementary rather than direct evidence.

Human sensory evaluation reflects integrated perceptual outcomes, whereas taste sensors quantify physicochemical interactions occurring at lipid/polymer membrane interfaces [99,100]. Although human taste perception involves receptor activation and downstream intracellular signaling pathways such as PLCβ2-mediated calcium mobilization, taste sensor outputs provide complementary physicochemical information that can be correlated with sensory responses [99,100]. The reported correlations between sensor responses and human bitterness scores further support the utility of sensor-based evaluation as a surrogate indicator of peripheral bitterness modulation during pharmaceutical development, although such correlations do not directly establish the underlying biological mechanisms.

At the central level, gustatory information is transmitted from the nucleus of the solitary tract to higher-order brain regions including the thalamus, insular cortex, and orbitofrontal cortex, where sensory integration and hedonic evaluation occur [101,102,103]. Dynamic interactions within these neural pathways contribute to modulation of perceived taste intensity and palatability beyond peripheral receptor activation [101,102,103].

Perceptual and affective factors also influence bitterness perception. Umami taste is associated with positive hedonic responses and activation of reward-related neural circuits [102,103,104]. Such effects may contribute to attenuation of aversive responses to bitter stimuli and partially explain interindividual variability in bitterness perception [102,103,104]. In addition, genetic polymorphisms in taste receptors and prior sensory experience further influence taste responses [102,103,104].

Temporal dynamics additionally affect taste integration. Differences in onset, persistence, and release kinetics among tastants can alter overall perceptual outcomes, particularly in complex pharmaceutical formulations containing multiple taste-related compounds [103,105,106]. In this context, integration of dissolution testing with electronic taste sensing provides a useful framework for evaluating dynamic changes in bitterness under conditions that more closely mimic oral administration.

Taken together, current evidence from mechanistic studies, human sensory evaluations, and sensor-based investigations supports the view that umami-mediated bitterness suppression may involve multiple levels of taste processing, including peripheral signaling, perceptual integration, and central modulation. However, the relationships among these different levels of evidence remain largely conceptual and have not yet been established through direct experimental evidence. In addition, independent validation by other research groups and evaluation using structurally diverse APIs will be important to determine the general applicability of umami-mediated bitterness suppression in pharmaceutical development. This perspective is broadly consistent with current concepts of sensory integration, in which taste perception emerges from interactions across neural, perceptual, and behavioral domains [101,102,103,104]. Recent advances in sensory neuroscience and flavor integration research further support the view that gustatory perception reflects dynamic integration of multisensory and affective signals rather than isolated receptor-driven events [103,105,106].

As summarized in Table 2, each bitterness evaluation method possesses distinct strengths and challenges. Human sensory testing serves as the gold standard, though constrained by ethical and safety issues, whereas cell assays offer high receptor specificity despite low throughput. In contrast, the BT0 taste sensor enables quantitative, high-throughput screening, providing a highly efficient approach. However, its scope is currently limited to peripheral interactions, necessitating complementary methods for comprehensive evaluation.

The convergence of human sensory outcomes and electronic taste sensor measurements provides complementary evidence that may support the development of a translational framework linking receptor-level observations, formulation performance, and patient-relevant palatability. Such a framework may facilitate more rational design of palatable pharmaceutical products while providing a conceptual basis for future validation of sensor-guided taste modulation strategies [101,102,103,104,105,106,107].

## 8. Future Perspectives and Clinical Implications

Despite substantial progress in understanding umami-mediated bitterness suppression, several challenges remain before its broad application in pharmaceutical formulation design can be achieved [108,109]. Future development will require closer integration of evidence from molecular studies, sensory evaluation, formulation science, and predictive analytical approaches while further clarifying the relationships among these different levels of evidence.

Human sensory evaluation remains essential for assessing overall taste perception; however, the results are influenced by subjective factors and interindividual variation. To complement sensory testing, electronic tongue technologies have been developed as objective tools for the quantitative assessment of taste qualities in the periphery [109]. Furthermore, validation studies have demonstrated the reliability and reproducibility of these systems, supporting their application in pharmaceutical taste evaluation and taste-masking research [110]. Regarding future prospects and clinical applications, establishing a clear correlation between human sensory evaluations and predictions from e-tongues or taste sensors remains difficult, largely because the human tongue exhibits cross-sensitivity to multiple taste stimuli. Nevertheless, BT0 and related taste sensors may provide useful surrogate measures of peripheral physicochemical responses associated with bitterness suppression, although their outputs should be interpreted together with human sensory and mechanistic evidence.

From a translational perspective, umami compounds such as MSG and 5′-ribonucleotides remain attractive due to their safety, food compatibility, and ability to modulate bitterness across diverse drug compounds [108,111]. Indeed, in Japan, MSG is already utilized in clinical and commercial formulations, as evidenced by its explicit inclusion as an inactive ingredient in the package inserts of oral morphine solutions [112] and various over-the-counter (OTC) internal medicines [113] to improve palatability.

To achieve rational formulation design that properly leverages such umami-mediated bitterness suppression, alternative evaluation methods are indispensable. In this regard, utilizing electronic taste sensors—which may serve as a valuable alternative to human sensory panels in specific applications—represents a highly promising approach. Although electronic taste sensors provide objective and reproducible measurements, broader regulatory and industrial acceptance will require further standardization and validation against human sensory outcomes [110,111]. From an industrial and regulatory perspective, the integration of novel bitterness suppression strategies into commercial pharmaceutical products faces several practical bottlenecks. Although electronic taste sensors and data-driven approaches offer high-throughput, objective evaluations, the lack of standardized regulatory guidelines for sensor validation against human sensory panels remains a major hurdle for regulatory approval. For instance, demonstrating the consistency, long-term stability, and bioequivalence of formulations utilizing umami compounds as functional taste-masking excipients requires rigorous quality control and clear justification of their safety limits across diverse patient populations. Overcoming these regulatory hurdles will necessitate establishing internationally accepted standards for predictive taste assessment tools, which would streamline formulation optimization and facilitate the commercial development of palatable oral dosage forms.

Recent advances in machine learning and data-driven formulation design provide new opportunities for optimizing bitterness suppression through integration of chemical descriptors, sensor outputs, and sensory data [111,114,115]. Emerging experimental platforms, including receptor-based assays and taste organoid models, further complement these approaches by enabling more physiologically relevant investigations of taste signaling and modulation [115].

Future studies should establish predictive frameworks that integrate physicochemical, experimental, computational, and sensory data, while also distinguishing direct experimental evidence from conceptual or inferential models of taste modulation.

To address such translational bottlenecks, establishing a critical and comprehensive evaluation framework is necessary. Currently, a major limitation of this field is the reliance on findings generated primarily by a few specialized groups. Independent replication by external research teams, along with systematic evaluations using structurally diverse APIs, will be critical to rigorously test the boundaries and generalizability of umami-mediated bitterness suppression.

Expansion to pediatric, geriatric, and dysgeusia-associated populations will also be essential for clinical translation. Overall, current evidence suggests that umami-mediated bitterness suppression is evolving from an empirical taste-masking concept toward a mechanistically informed and quantitatively supported strategy, although further studies are needed to establish direct mechanistic links among receptor-level events, sensory perception, and analytical measurements.

From a pharmaceutical perspective, umami-mediated bitterness suppression possesses several features that distinguish it from conventional taste-masking strategies. Physical and chemical approaches primarily reduce the exposure of APIs to bitter taste receptors through formulation-dependent barriers, whereas receptor-oriented antagonists generally target limited subsets of TAS2R receptors. In contrast, current evidence suggests that umami compounds may influence bitterness through multiple hierarchical levels of gustatory processing—including peripheral receptor interactions, intracellular signaling pathways, local taste-bud networks, perceptual mixture effects, and higher-order sensory integration—although the relative contribution of each level remains to be fully clarified.

Importantly, umami substances are widely used food ingredients with established safety profiles and favorable formulation compatibility. In addition to reducing bitterness, they may contribute to overall taste balance and palatability, potentially improving the sensory quality of oral medicines beyond bitterness masking alone. Although direct comparisons with sweeteners, receptor antagonists, and physicochemical masking systems remain limited, current evidence suggests that umami-mediated taste modulation represents a promising complementary strategy that could be integrated with existing formulation technologies. Such a multilevel conceptual framework may provide a useful basis for improving patient acceptability of bitter oral medications while guiding future mechanistic and translational studies.

## 9. Conclusions

Bitterness remains a significant challenge in pharmaceutical development because it directly affects patient adherence and therapeutic outcomes, particularly in pediatric and geriatric populations. Although conventional taste-masking strategies can reduce bitterness to some extent, they are often formulation-dependent and do not fully address the biological mechanisms underlying taste perception.

Umami compounds provide a unique approach to bitterness suppression by modulating taste perception through multiple mechanisms, including peripheral taste interactions, perceptual mixture effects, and higher-order neural and sensory processing.

Current evidence suggests that these multiple contributing processes may collectively contribute to umami-mediated bitterness suppression, distinguishing umami-based modulation from conventional physicochemical masking approaches and supporting a broader view of taste perception as a multilevel sensory integration phenomenon.

Recent advances in electronic taste-sensing technologies have enabled objective and reproducible evaluation of bitterness and its modulation by umami substances. When combined with multivariate analysis and predictive data-driven approaches, these systems offer a quantitative framework linking physicochemical properties, sensor responses, and human sensory perception. Notably, sensor-based evaluations—such as those utilizing the BT0 membrane sensor—provide a useful approach for evaluating the peripheral component of umami-mediated bitterness suppression and may preferentially reflect interactions associated with specific bitter tastants.

Collectively, current evidence supports the concept that umami-mediated bitterness suppression involves multiple levels of taste processing; however, the relationships among these processes remain incompletely understood and require further experimental validation. Umami-mediated bitterness suppression therefore represents a promising framework for the rational design of more palatable oral medicines and may ultimately contribute to improved patient acceptability, medication adherence, and clinical outcomes.

Future integration of receptor-based biology, electronic taste sensing, and data-driven formulation design may help establish umami-mediated taste modulation as a practical platform for pharmaceutical development.

## Figures and Tables

**Table 2:** Strengths and challenges of evaluation methods for umami-induced bitterness suppression.

Method	Strength	Challenge (Weakness)
Human sensory	Gold standard	Subjective, limited sample size
Cell assay	Receptor specificity	High cost & specialized setup
Taste sensor (BT0)	Quantitative & high-throughput	Limited to peripheral interactions

## Data Availability

Data are available upon request.

## Supplementary Materials

The following supporting information can be downloaded at https://www.mdpi.com/article/10.3390/s26165073/s1.
