# Supplementary material for: Taste Modulation by Umami Compounds: Mechanisms, Sensor-Based Evaluation, and Pharmaceutical Applications in Bitterness Suppression
Source: Sensors (Basel). 2026 Aug 10;26(16):5073. doi: 10.3390/s26165073 (PMC13517633; doi:10.3390/s26165073)
Supplement: Supplementary file 1 [file sensors-26-05073-s001.zip › sensors-4415368-supplementary.pdf]

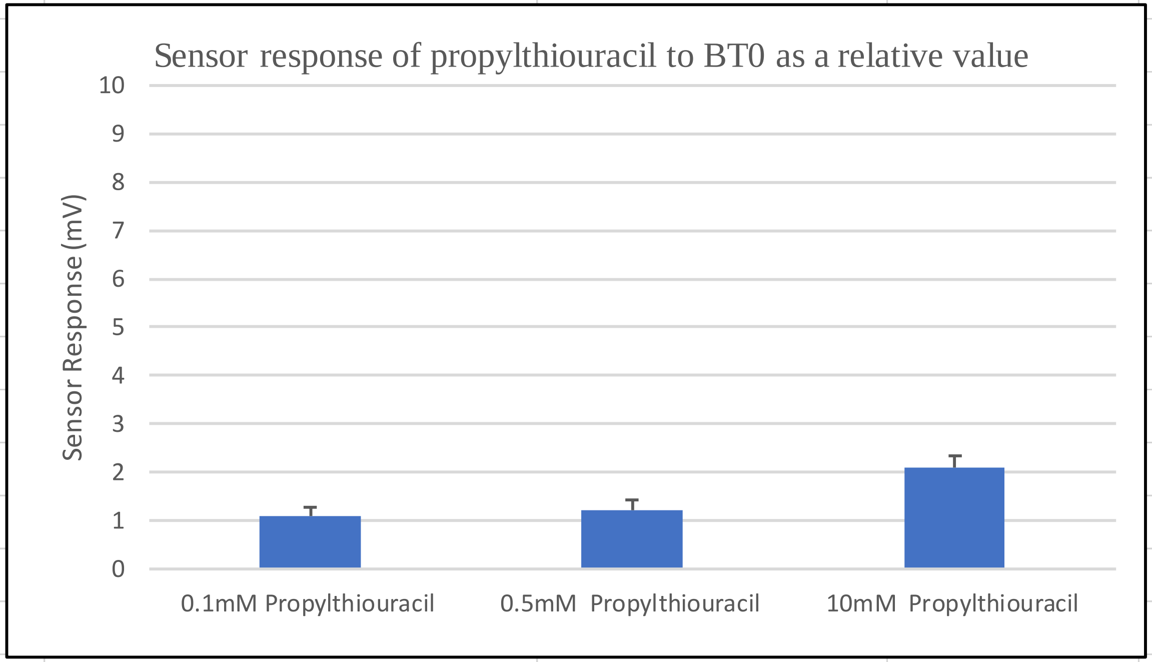

Figure S1. S ensor response of 0.1. 0.5, and 10mM propylthiouracil to BT0 as a relative value. The sample solution were prepared by reference solution (30 mM KCl, 0.3 mM tartaric acid). The sensor measurement was performed according to the previous article [31].
